# Hydrothermal extraction, a promising method for concentrating phenolic antioxidants from red osier dogwood (*Cornus stolonifer*) leaves and stems

**DOI:** 10.1016/j.heliyon.2020.e05158

**Published:** 2020-10-08

**Authors:** Franklin B. Apea-Bah, Dagmara Head, Robert Scales, Ron Bazylo, Trust Beta

**Affiliations:** a Department of Food and Human Nutritional Sciences; Faculty of Agricultural and Food Sciences, University of Manitoba, Fort Garry Campus, Winnipeg, Manitoba R3T 2N2, Canada; b Food Development Centre, Portage la Prairie, Manitoba, R1N 3J9, Canada; c Red Dog Enterprises Ltd., Swan River, MB, R0L 1Z0, Canada; d Manitoba Agriculture, Dauphin, MB, Canada

**Keywords:** Food science, Red osier dogwood, *Cornus stolonifer*, Hydrothermal extraction, Phenolic acids, Flavonoids, Antioxidant properties

## Abstract

Red osier dogwood (ROD) (*Cornus stolonifer*) is a popular ornamental shrub in most parts of North America. It has a record of ethnopharmacological uses by native North Americans. With increasing awareness about the health benefits of natural antioxidants, efforts are needed to develop methods for producing plant-based antioxidants as sources of nutraceuticals or functional food ingredients. This study aimed at establishing an optimum temperature for hydrothermal extraction of phenolic compounds from the leaves and stems of ROD. Spray-dried extracts obtained from hydrothermal extraction at four different temperatures, as well as their raw materials and spent residue were analyzed for moisture content and water activity. The samples were extracted with organic solvent and their total phenolic content, phenolic composition and antioxidant activity were also determined. Moisture content was below 10% and the water activity was below 0.6 inclusive, which are recommended for storing dry plant products. Glucogallic acid, ellagic acid, rutin, quercetin 3-*O*-malonylglucoside and quercetin were the phenolic compounds identified in all the samples. Rutin was the predominant compound. As expected, all the spray-dried extracts had higher phenolic content and antioxidant activity than the raw materials and spent materials. Among the temperatures studied, 98 °C was the most effective in extracting the phenolic compounds. The spray-dried extracts may find application in high-value antioxidant-rich products such as functional food ingredients and nutraceuticals. The spent materials retained a considerable amount of phenolic antioxidants and can therefore be useful in preparing antioxidant-rich animal feed.

## 1 Introduction

Red osier dogwood (ROD) (*Cornus stolonifer*) is a shrub belonging to the *Cornus* genus and native to North America where it grows as an ornamental plant. It is therefore used for landscaping due to its beautiful flowers which blossom during summer or early fall (Isaak et al., 2013). Most research has focused on fruits of the *Cornus* genus, which are popular in North America, Europe and Asia (Isaak et al., 2013). Edible fruits belonging to this genus are rich in phenolic antioxidants of the classes flavonols (kaempferol, quercetin and their glycosides) (Pawlowska et al., 2010) and anthocyanins (cyanidin, delphinidin, pelargonidin and their glycosides) (Du et al., 1974; Vareed et al., 2006). The leaves and stems of ROD are rich in phenolic antioxidants of the classes, phenolic acids (gallic and ellagic acids) (Isaak et al., 2013), flavonols (kaempferol, quercetin and their glycosides) (Isaak et al., 2013), flavan-3-ols (catechin and epicatechin), anthocyanins (cyanidin) (Isaak et al., 2013) and phenylethanoids (tyrosol) (Wei et al., 2018). The ethnopharmacological uses of ROD fruits, leaves, stems and roots against several ailments have been well documented for indigenous North Americans (Smith, 1929).

Epidemiological studies suggest a positive relationship between consumption of dietary phenolic compounds and reduction in the incidence of chronic diseases of lifestyle such as cardiovascular and coronary heart diseases, type II diabetes and various forms of cancer (Hooper et al., 2008). The antioxidant properties of phenolic compounds enable them to scavenge reactive oxygen and nitrogen species which if not modulated in the human body, can damage physiological molecules resulting in sustained inflammation and consequent debilitating diseases (Apea-Bah, 2014). Dietary phenolic compounds can therefore complement the natural antioxidant defense system in the body and provide protection against oxidative stress-related diseases. While ROD berries can provide nourishment for both humans (Isaak et al., 2013) and animals (Timmermann and McNicol, 1988), their leaves and stems can also be harnessed for the production of phenolic antioxidant-rich functional food ingredients and nutraceuticals.

Phenolic compounds from dogwood and other plant materials are extracted mostly using alcohols such as methanol (McCune and Johns, 2002), ethanol (Nair and Rudloff, 1960) and their acidified derivatives (Isaak et al., 2013), or aqueous acetone (McArthur et al., 1993). These polar organic solvents have been established as the most suitable for extraction of phenolic compounds (Liyana-Pathirana and Shahidi, 2005), which are polar organic molecules. The health, safety and flammable hazard concerns associated with the use of these organic solvents challenge their usefulness in food applications. Although water is a poor extraction solvent for phenolic compounds (Al-Farsi and Lee, 2008), it is a safer alternative both from health and flammability perspectives. Water has been applied in the extraction of phenolics from marama bean (*Tylosema esculentum*) seed coat (Shelembe et al., 2012) and sorghum (*Sorghum bicolor*) bran (Shelembe et al., 2014). Acidification of the water increased its phenolic extraction power from sorghum bran (Shelembe et al., 2014).

Besides polarity of solvent, other factors that are important to phenolic extractability are the extraction temperature and extraction time. High temperatures may favour extraction of thermally stable molecules by mobilizing them and enhancing their solubility in the solvent, but may also cause decomposition of heat labile phenolic compounds (Liyana-Pathirana and Shahidi, 2005; Wettasinghe and Shahidi, 1999). In a plant matrix containing a cocktail of phenolic compounds, the overall effect of extraction at high temperatures will depend on the relative proportions of heat stable and labile compounds. Skotti et al. (2014) demonstrated that aqueous extracts from some Greek medicinal and aromatic plants had higher total phenolic content and antioxidant activity when extracted at a higher temperature (85 °C) than when extraction was done at room temperature. Sharma et al. (2015), on the other hand, demonstrated that when onion powder was heated up to 120 °C for 30 min, its total phenolic content and concentrations of quercetin and quercetin glucosides increased.

The aim of this research was to identify the most suitable temperature for preparation of a powdered aqueous phenolic extract from red osier dogwood leaves and stems. This product could have applications as functional food ingredient or nutraceutical ingredient due to its high content of phenolic compounds.

## 2 Materials and methods

### 2.1 Reagents

The phenolic standards gallic acid, ellagic acid, quercetin and rutin hydrate, as well as 6-hydroxy-2,5,7,8-tetramethylchroman-2-carboxylic acid (Trolox), 2,2′-azino-bis(3-ethylbenzothiazoline-6-sulfonic acid) (ABTS), 2,2′-azobis(2-amidinopropane) dihydrochloride (AAPH), 2,2-diphenyl-1-picrylhydrazyl (DPPH) and fluorescein disodium salt were purchased from Sigma-Aldrich Chemical Co. (St. Louis, MO, USA). HPLC-grade methanol, acetic acid, formic acid, LCMS-grade acetonitrile and all other chemicals were procured from ThermoFisher Scientific (West Palm Beach, FL, USA).

### 2.2 Source of samples and preparation of spray-dried ROD extracts

ROD samples which were kindly donated by Red Dog Enterprise Ltd., comprised leaves and stems that were freshly harvested from Swan River (Manitoba, Canada), air-dried and pulverized into tiny particles. They were divided into 5 groups and extracted under the following hydrothermal conditions: a) 75 °C for 1 h; b) 75 °C for 2 h; c) 85 °C for 1 h; d) 95 °C for 1 h; e) 98 °C for 1 h. The extraction conditions were selected following previous preliminary trials. The extraction procedure involved steeping of samples in water, solid separation, clarification/purification, concentration and spray drying.

The samples were steeped in an open kettle (Cleveland MKDL150, 500 L, Concord, ON, Canada) at a ratio of 1:10 of sample (40 kg) to water (400 L), under mechanical agitation (34 rpm), at the specified temperatures and durations. After steeping, the hot slurry was sieved through a 38 μm Kason sifter (K24 3 SS, Vibroscreen) to remove coarse solids. The residue was passed through a belt press (Frontier Technology Inc., Allegan, MI, USA) to squeeze out the remaining liquid, which was again passed through the Kason sifter. The sieved liquid was clarified using a combination of polishing disc stack centrifuge (SB7-01-076, GEA Westfalia, Burlington, ON, Canada) and bag filter with pore size of 1.5 μm.

The filtrate obtained from the clarification process was passed through a membrane filtration system (Model R, GEA Pilot Plant Unit, Hudson, WI, USA) equipped with reverse osmosis (RO) membrane filters to concentrate the extract. The RO retentate obtained was spray-dried at an inlet temperature of 235 °C, an outlet temperature of 93 °C, and a feed temperature of 20–30 °C. The resulting powdered extract was collected from the cyclone collection vessel at 15 min intervals, allowed to cool, and packed in food-grade lined plastic pail.

The spent material or residue remaining after extraction was frozen at -20 °C and freeze-dried. Portions of the raw materials (pulverized leaves and stems) and freeze-dried spent materials were milled into powder. The spray-dried extracts and milled samples were kept at -20 °C for phenolic extraction and analyses. In total, 14 samples were prepared.

### 2.3 Moisture content

Moisture content of the samples was determined by drying one gram of the milled sample in an oven at 105 °C to constant weight. The moisture content was then calculated from the loss in weight and expressed as a percentage relative to the initial weight (AOAC, 2000).

### 2.4 Water activity

Water activity of the samples was determined using a portable water activity meter (Novasina LabSwift-aw™, Cole-Parmer, Montreal, QC, Canada) (Fernandes et al., 2017).

### 2.5 Extraction of phenolic compounds

Extraction of the constituent phenolic compounds was done according to the method of Isaak et al. (2013) with modification. A 0.5 g of spray-dried extract or milled sample (raw and freeze-dried spent materials) was weighed into a 15 mL capacity Falcon™ conical centrifuge tube and extracted twice with 6 mL of 2% (v/v) formic acid in HPLC-grade methanol under sonication for 1 h. After each extraction, the suspension was centrifuged at 7000 × *g* for 10 min and the supernatant removed. The pooled supernatant was filtered through 0.2 μm syringe filter and used for analyses.

### 2.6 Phenolic composition

The phenolic composition of the samples was determined based on a modification of the method of Qiu et al. (2009) using a Waters Alliance 2695 high performance liquid chromatograph (HPLC) (Milford, MA, USA) equipped with a quaternary pump, a Waters 2996 photodiode array detector (Milford, MA, USA), and a Waters 717 Plus autosampler. The HPLC was coupled to a Micromass quadrupole time-of-flight mass spectrometer (Q-TOFMS) (Waters, Milford, MA, USA). The extract (10 μL) was injected onto an Accucore aQ, 100 × 3 mm, 2.6 μm column (Thermo Scientific, West Palm Beach, FL, USA) and eluted with a binary mobile phase comprising solvent A (0.1% aqueous acetic acid) and solvent B (0.1% acetic acid in acetonitrile), at a flow rate of 0.5 mL/min. The constituent phenolic compounds were separated using the following linear gradient: 0–2.5 min, 5–10% B; 2.5–7.5 min, 10–15% B; 7.5–10 min, 15–20% B; 10–15 min, 20–25% B; 15–20 min, 25–40% B; 20–22.5 min, 40-10% B; 22.5–25 min, 10-5% B. Column temperature was set at 35 °C and sample temperature was set at 15 °C. The Q-TOFMS was calibrated using sodium iodide for the negative electrospray ionization (ESI) mode over a mass range of 100–1500 amu. The compounds were identified by comparing their retention times and UV and mass spectral characteristics with that of authentic standards where available, and compounds reported in literature (mainly for glucogallic acid and quercetin 3-*O*-malonylglucoside, for which no authentic standards were available). Glucogallic acid was quantified based on the peak area at a wavelength of 280 nm using an authentic gallic acid external standard curve, while the other phenolic compounds were identified at 350 nm using standard curves plotted with their corresponding authentic standards. Quercetin 3-*O*-malonylglucoside was quantified as quercetin equivalent. The results were expressed as milligram per 100 g (mg/100 g) of sample, on dry weight basis. Full mass spectra were recorded in negative ESI mode using a capillary voltage of 1.2 kV and a cone voltage of 45 V. The desolvation gas and ion source temperatures were set at 250 °C and 120 °C, respectively. The MS/MS spectra were acquired by using argon gas and a collision energy of 30 V. MassLynx v. 4.1 software (Waters, Milford, MA, USA) was used for data acquisition.

### 2.7 Total phenolic content

Total phenolic content (TPC) of the appropriately diluted extracts (spray-dried extracts were diluted 200 times while the raw and spent materials were diluted 100 times with methanol) was determined according to the method of Apea-Bah et al. (2016) using a 96-well microplate reader (ELX800, BioTek Instruments, Winooski, VT, USA). The method involved reacting 18.2 μL of diluted extract with 36.4 μL of 10% (v/v) aqueous Folin-Ciocalteu reagent and 145.4 μL of 700 mmol/L sodium carbonate in the dark at room temperature (20–22 °C) for 2 h. Absorbance of the mixture was then read at 750 nm. Gallic acid (0.025–0.150 mg/mL) was used as a standard and results were expressed as gram gallic acid equivalents per 100 g (g GAE/100 g) sample, dry weight basis.

### 2.8 DPPH radical scavenging capacity

The method of Brand-Williams et al. (1995) was modified for assay in the 96-well microplate format. This involved adding 195 μL of 60 μmol/L DPPH methanolic solution to 5 μL of appropriately diluted extract (spray-dried extracts were diluted 400 times with methanol while the raw and spent materials were diluted 100 times with methanol). The reaction mixture was incubated for 60 min in the dark and the absorbance was read at 515 nm. Trolox (25–800 μmol/L) was used as a standard and results were expressed as millimole Trolox equivalents per 100 g (mmol TE/100 g) sample, dry weight basis.

### 2.9 Trolox equivalent antioxidant capacity

The method described by Apea-Bah et al. (2016) for determination of Trolox equivalent antioxidant capacity (TEAC) was used. For the analysis, the spray dried extracts were diluted 400 times while the raw and spent materials were diluted 100 times with methanol. The results were expressed as mmol TE/100 g sample, dry weight basis.

### 2.10 Oxygen radical absorbance capacity

The oxygen radical absorbance capacity (ORAC) of the appropriately diluted extracts (spray-dried extracts were diluted 4000 times while the raw and spent materials were diluted 1000 times with 75 mmol/L potassium phosphate buffer, pH 7.4) was determined using a modification of the method of Qiu et al. (2010). The modification involved reacting 150 μL of 0.816 nmol/L fluorescein with 25 μL of 153 mmol/L AAPH in the presence of 25 μL of diluted extract, blank (75 mmol/L potassium phosphate buffer, pH 7.4) or Trolox standard (6.25–50 μmol/L), and measuring the fluorescence decay (area under curve) at 37 °C over 50 min. ORAC values were expressed as mmol TE/100 g sample, dry weight basis.

### 2.11 Statistical analysis

Results from all analyses were presented as means ± standard deviations of at least triplicates. Two-way analysis of variance was used to determine the effects of hydrothermal treatment and replication on dependent variables. Fisher's least significant difference was used to compare means that significantly (p < 0.0001) differed from each other. Principal components analysis was performed to evaluate the association between the response variables. Statistica 10 (StatSoft Inc., Tulsa, OK, USA) was used for statistical analysis.

## 3 Results and discussion

### 3.1 Phenolic composition

**Figure 1:**
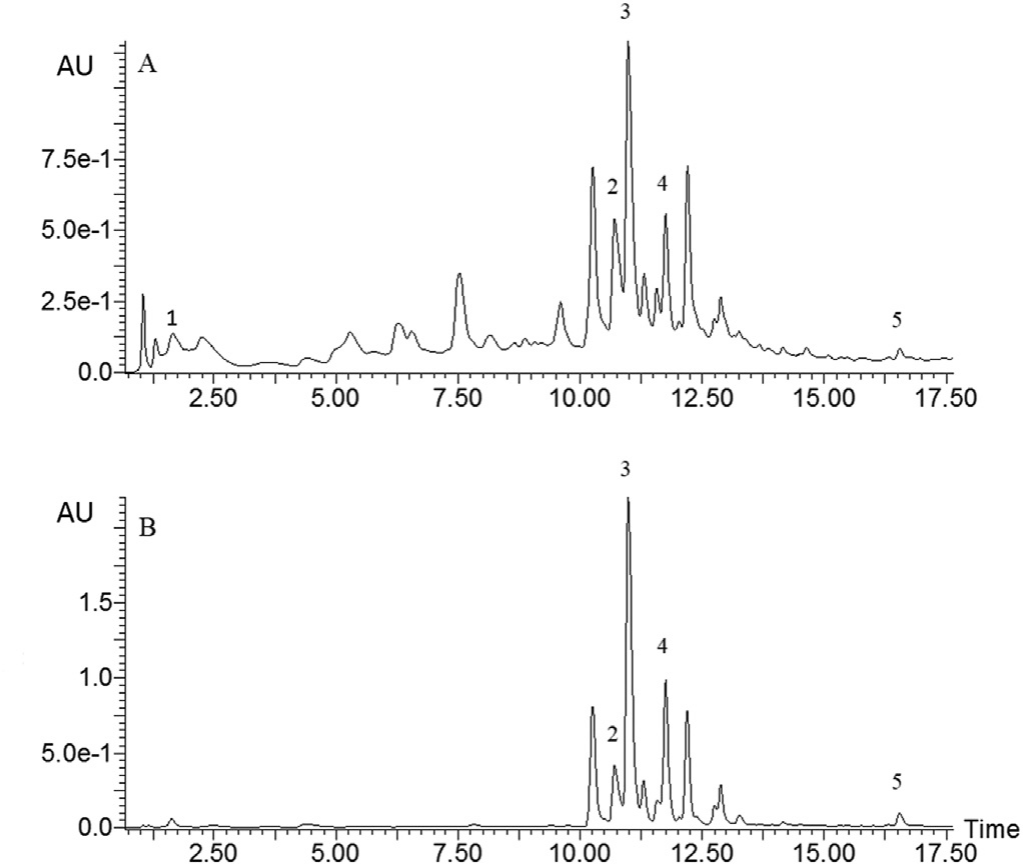
Chromatograms of phenolic compounds identified in red osier dogwood leaves and stems obtained at 280 nm (A) and 350 nm (B). Key: 1 = glucogallic acid; 2 = ellagic acid; 3 = rutin; 4 = quercetin 3-*O*-malonylglucoside; 5 = quercetin.

**Figure 2:**
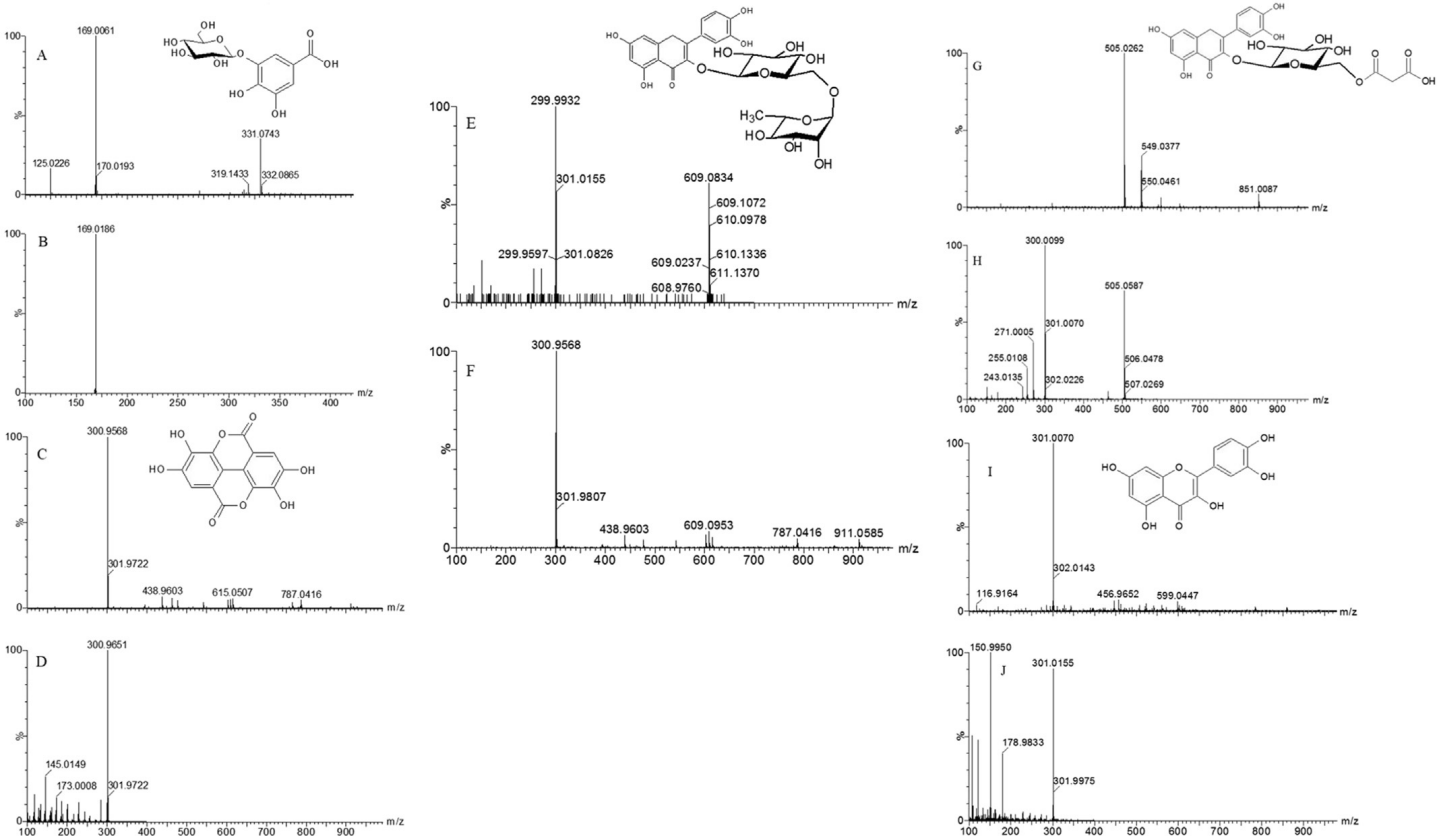
Mass spectra and chemical structures of phenolic compounds identified in red osier dogwood leaves and stems. A: mass spectrum and chemical structure of precursor ion for glucogallic acid; B: mass spectrum of product ion for glucogallic acid; C: mass spectrum and chemical structure of precursor ion for ellagic acid; D: mass spectrum of product ion for ellagic acid; E: mass spectrum and chemical structure of precursor ion for rutin; F: mass spectrum of product ion for rutin; G: mass spectrum and chemical structure of precursor ion for quercetin 3-*O*-malonyl glucoside; H: mass spectrum of product ion for quercetin 3-*O*-malonyl glucoside; I: mass spectrum and chemical structure of precursor ion for quercetin; J: mass spectrum of product ion for quercetin.

Five phenolic compounds were identified based on their retention times, UV and mass spectral data which were compared with those of authentic standards and published reports. Sample chromatograms obtained at 280 nm and 350 nm are shown in Figure 1, while the corresponding mass spectra for their precursor and product ions are shown in Figure 2.

The compounds identified comprised two phenolic acids: glucogallic acid (gallic acid glucoside) and ellagic acid, and three flavonoids: rutin, quercetin 3-*O*-malonylglucoside and quercetin (Table 1). Rutin was the predominant compound in all the samples, followed by glucogallic acid. Quercetin was the lowest in concentration. These findings are in agreement with the report of Isaak et al. (2013). However, Isaak et al. (2013) reported gallic acid but not glucogallic acid, using HPLC analysis which bases identification on the retention time and UV-visible spectra of the compound. The current research identified glucogallic acid based on its wavelength for maximum absorption (λmax) and mass spectra (Table 1, Figure 2), in comparison with similar compounds reported in other plants (Abdallah et al., 2019; Liu et al., 2020; Pan et al., 2019). To the best of our knowledge, this is the first time gallic acid has been identified as a glucoside in red osier dogwood. The glycosylation is essential in enhancing its solubility in water (Pan et al., 2019). Although, the position of glycosylation could not be determined, the most reported isomer in plants is 3-glucogallic acid (Ma et al., 2021; Nayeem et al., 2016). It is therefore presumable that 3-glucogallic acid was tentatively identified in red osier dogwood in the current study.

**Table 1:** Peak number, retention time, UV absorption maxima, mass spectral data and chemical formulae of phenolic compounds from red osier dogwood leaves and stems.

No.	R_T_, min	λmax, nm	[M-H]^-^	MS/MS	Chemical formula	Compound
1	1.32	280	331	169	C_13_H_16_O_10_	Glucogallic acid
2	10.64	280, 350	301	301	C_14_H_6_O_8_	Ellagic acid
3	10.98	280, 364	609	301, 300	C_27_H_30_O_16_	Rutin
4	11.75	256, 354	549	300, 301, 505	C_24_H_22_O_15_	Quercetin 3-*O*-malonylglucoside
5	16.55	278, 354	301	151, 301,178	C_15_H_10_O_7_	Quercetin

Key: No. = peak number; R_T_ = retention time; λmax = UV wavelength at maximum absorption; [M-H]^-^ = precursor ions; MS/MS = product ions. Glucogallic acid was identified at 280 nm while all other compounds were identified at 350 nm.

It is worth noting that quercetin was present in higher amount mostly as glycosides giving rise to rutin and quercetin 3-*O*-malonylglucoside. While quercetin is absorbed in the small intestine, rutin is absorbed mostly in the colon after fermentation and consequent deglycosylation by the colonic microbiota. Rutin absorption is therefore slower than that of quercetin (Manach et al., 1997). Therefore, rutin is an important antioxidant both in the upper and lower gastrointestinal regions. After absorption, quercetin is detected in human plasma mainly in conjugated forms such as glucuronide conjugates with or without methylation, and not as aglycone (Manach et al., 1998; Graefe et al., 2001). Since the rate of excretion of quercetin metabolites from systemic circulation is rather low, high plasma concentrations can easily be maintained with regular consumption of quercetin- or rutin-containing diet (Manach et al., 1997).

Table 2 shows the phenolic composition of the ROD samples. Glucogallic acid content ranged from 115-304 mg/100 g in the raw materials, 1447–2003 mg/100 g in the spray-dried extracts and 131–683 mg/100 g in the spent material. The higher concentration of glucogallic acid in the spent material may indicate the low extraction efficiency of the method for glucogallic acid in the original raw material, since extraction was done only once. The hydrothermal treatment caused tissue disruption in the spent material which probably increased the extractability of the remaining glucogallic acid in the freeze-dried spent material. This may be supported by the practice of decoction (a hydrothermal process) to extract polyphenols from medicinal plants (Severi et al., 2009). Ellagic acid was detected only in the spray-dried extracts and its content ranged from 185-695 mg/100 g. Rutin content ranged from 754-1189 mg/100 g in the raw materials, 1214–2713 mg/100 g in the spray-dried extracts, and 336–530 mg/100 g in the spent materials. The rutin content recorded in the raw materials was in agreement with values reported by Isaak et al. (2013). Quercetin 3-*O*-malonylglucoside content ranged from 185-353 mg/100 g in the raw materials, 192–418 mg/100 g in the spray-dried extracts, and 24–50 mg/100 g in the spent materials. Quercetin content ranged from 0.7-5 mg/100 g in the raw materials, 4–13 mg/100 g in the spray-dried extracts, and 6–11 mg/100 g in the spent materials. The higher level of quercetin in spent material compared to raw material is likely due to the low extraction efficiency of the method and low extraction capacity of water for the compound in the latter, and the tissue disruption of the spent material by hydrothermal treatment, as described earlier for glucogallic acid. In comparison, Isaak et al. (2013) recorded quercetin values only after acid hydrolysis of the constituent rutin and other glycosides. This confirms that quercetin is present in ROD in the aglycone form only at low levels. It is noteworthy, however, that quercetin in the aglycone form, is only sparingly soluble in water, and therefore exists in most plants as glycosides.

**Table 2:** Phenolic composition of hydrothermal extracts from red osier dogwood leaves and stems.

Sample	Glucogallic acid	Ellagic acid	Rutin	Quercetin malonyl glucoside	Quercetin	Total quantified phenolics content
Extraction at 75^o^C						
Raw material, 1 h	239.77 ± 2.42^abc^	nd	892.00 ± 4.35^f^	233.27 ± 0.35^g^	4.41 ± 0.07^e^	1602.88 ± 7.07^de^
Spray dried extract, 1 h	1446.82 ± 9.76^e^	240.97 ± 6.80^b^	1932.44 ± 78.87^k^	418.15 ± 12.97^l^	10.05 ± 3.4^k^	4550.19 ± 98.88^i^
Spray dried extract, 2 h	1546.82 ± 13.33^ef^	255.28 ± 11.67^c^	2196.52 ± 9.01^l^	330.02 ± 0.66^j^	13.11 ± 0.07^o^	4825.03 ± 6.57^j^
Spent material, 1 h	130.72 ± 1.76^a^	nd	397.25 ± 8.03^b^	50.18 ± 0.20^c^	10.15 ± 0.12^k^	675.81 ± 9.60^a^
Spent material, 2 h	148.17 ± 2.28^ab^	nd	530.42 ± 8.35^c^	41.50 ± 1.45^b^	10.69 ± 0.06^m^	827.68 ± 5.49^a^
Extraction at 85^o^C						
Raw material, 1 h	215.52 ± 0.36^abc^	nd	753.69 ± 1.93^d^	291.32 ± 2.04^h^	2.66 ± 0.01^c^	1490.43 ± 6.28^cd^
Spray dried extract, 1 h	1618.25 ± 22.09^fg^	185.47 ± 1.82^a^	1214.03 ± 2.03^i^	235.09 ± 5.88^g^	4.40 ± 0.11^e^	3616.11 ± 22.61^h^
Spent material, 1 h	330.02 ± 0.60^c^	nd	336.12 ± 0.22^a^	38.65 ± 0.23^b^	6.05 ± 0.04^g^	791.71 ± 1.25^a^
Extraction at 95^o^C						
Raw material, 1 h	228.75 ± 1.75^abc^	nd	995.06 ± 4.33^g^	223.11 ± 1.67^f^	5.18 ± 0.08^f^	1668.68 ± 7.25^ef^
Spray dried extract, 1 h	2002.94 ± 396.24^h^	432.22 ± 7.10^e^	1637.54 ± 42.26^j^	232.36 ± 9.04^g^	8.67 ± 0.21^i^	4738.16 ± 375.66^j^
Spent material, 1 h	554.17 ± 6.69^d^	nd	427.31 ± 10.12^b^	23.55 ± 0.49^a^	9.27 ± 0.18^j^	1095.19 ± 13.89^b^
Extraction at 98^o^C						
Raw material, 1 h	304.04 ± 12.63^bc^	nd	1079.28 ± 18.85^h^	354.77 ± 9.52^k^	1.19 ± 0.01^b^	2113.21 ± 40.89^g^
Spray dried extract, 1 h	1682.25 ± 28.76^fg^	671.93 ± 6.83^f^	2712.91 ± 56.42^m^	313.21 ± 3.35^i^	10.20 ± 0.02^k^	6277.75 ± 63.29^k^
Spent material, 1 h	683.29 ± 7.3.3^d^	nd	500.33 ± 5.81^c^	26.78 ± 1.23^a^	8.27 ± 0.04^h^	1351.80 ± 9.82^c^

Results are presented as means ± standard deviation of triplicates. Glucogallic acid was expressed as gallic acid equivalents. Quercetin 3-*O*-malonylglucoside was expressed as quercetin equivalents. Results are expressed as mg/100 g sample, dry weight basis. Values in a column with same superscript letters are not significantly (p < 0.0001) different from each other.

Hydrothermal extraction of the raw materials at 75 °C for 1 h increased contents of glucogallic acid by 503% (6 fold), rutin by 117% (2 fold), quercetin 3-*O*-malonylglucoside by 79% (1.8 fold), and quercetin by 128% (2.3 fold), accompanied by the release of 241 mg/100 g of ellagic acid. Increasing the extraction time to 2 h at 75 °C, increased levels of ellagic acid by 7%, rutin by 14%, and quercetin by 30%. Following hydrothermal extraction, the spent material retained, on the average, 67% glucogallic acid, 54% rutin and 21% quercetin 3-*O*-malonylglucoside compared to the amounts present in the raw material. Quercetin was 2.6 times more extractable from the spent material than in the raw material using acidified methanol, probably due to plant tissue disruption during the hydrothermal extraction at 75 °C.

Hydrothermal extraction of the raw materials at 85 °C for 1 h, increased levels of glucogallic acid by 651% (8 fold), rutin by 61% (2 fold) and quercetin by 66% (2 fold), accompanied by the release of 185 mg/100 g of ellagic acid. The spent material retained 45% rutin and 13% quercetin 3-*O*-malonylglucoside, compared to amounts present in the raw material. Glucogallic acid and quercetin were 53% and 127%, respectively more extractable from the spent material than from the raw material using acidified methanol.

Hydrothermal extraction of the raw materials at 95 °C for 1 h, on the average, increased levels of glucogallic acid by 983% (8.8 fold), rutin by 83% (1.6 fold), and quercetin by 130% (1.7 fold), accompanied by the release of 404 mg/100 g of ellagic acid. However, the quercetin 3-*O*-malonylglucoside content in the extract was 5% lower than in the raw material. After hydrothermal extraction, the spent material retained 222% (3 fold) more of glucogallic acid and 114% more of quercetin than were extracted from the raw samples. It also retained 43% of rutin and 11% of quercetin 3-*O*-malonylglucoside relative to the amounts extracted in the raw material.

Hydrothermal extraction of the raw materials at 98 °C for 1 h increased contents of glucogallic acid by 453%, rutin by 151% and quercetin by 757%, accompanied by the release of 672 mg/100 g of ellagic acid. The spray-dried extract, on the average, contained 11% less of quercetin 3-*O*-malonylglucoside than the amount present in the raw material. The spent material contained 125% more glucogallic acid and 595% more quercetin contents than the raw material. It is clear from the results that at 85 °C and above, quercetin 3-*O*-malonylglucoside was the most thermally labile among the phenolic compounds identified.

Total phenolics concentration was computed as the sum of phenolic compounds identified in each sample. Increasing extraction time from 1 h to 2 h at 75 °C increased the total phenolics concentration in the spray-dried extracts by 6%. Increasing extraction temperature from 75 °C to 85 °C for the same 1 h duration caused a 26% decrease in total phenolics concentration of the spray-dried extract. The spray-dried extracts prepared at 95 °C for 1 h had 31% higher total phenolics concentration than those prepared at 85 °C for 1 h, and 4% higher total phenolics concentration than extracts prepared at 75 °C for 1 h. However, their total phenolics concentration was comparable to spray-dried extracts prepared at 75 °C for 2 h. The total phenolics concentration of spray-dried extracts prepared at 98 °C, 1 h was 39%, 31%, 75% and 34% higher than corresponding extracts prepared at 75 °C (1 h), 75 °C (2 h), 85 °C (1 h) and 95 °C (1 h) respectively.

Total phenolics concentration of spent material for samples extracted at 75 °C (1 h), 75 °C (2 h) and 85 °C (1 h) were all statistically comparable; however, they were all lower than values obtained at 95 °C (1 h) and 98 °C (1 h). Among all the spent materials, the ones from raw materials extracted at 98 °C (1 h) had the highest concentration. This suggests that the hydrothermal extraction process at 98 °C was the most effective in disrupting the plant tissues to release phenolic compounds for extraction. Phenolic acids and flavonoids are organic compounds and are therefore more soluble in organic solvents such as methanol, ethanol, acetone or their acidified and aqueous mixtures (Nair and Rudloff, 1960; Isaak et al., 2013; McCune and Johns, 2002; Liyana-Pathirana and Shahidi, 2005), than water alone (Al-Farsi and Lee, 2008). This explains the substantial phenolics retention in the spent materials. However, health safety concerns and hazards associated with volatile and flammable organic solvents may necessitate extraction using water. In that case, the spent material can further be processed into high-value products containing phenolic antioxidants, such as animal feed. Indeed, Wei et al. (2019) reported ROD to have better feed value than silage in high-grain diets for beef cattle, and improves immune status and antioxidant activity in finishing beef.

### 3.2 Moisture content, water activity, total phenolic content and antioxidant activity

Table 3 shows the moisture content, water activity, total phenolic content (TPC) and antioxidant activity of the dogwood samples. The moisture content of the raw materials, spray-dried extracts and freeze-dried spent materials ranged between 7.6-9.3%, 5.2–7.3% and 1.1–3.7% respectively. Therefore, on the average, the freeze-dried spent materials had the lowest moisture content while the raw materials had the highest moisture content. According to Clemenson et al. (2012), a maximum of 12% moisture content is usually set for herbs and spices to maintain their stability. This is in agreement with the report of Müller and Heindl (2006) for dried medicinal plants.

**Table 3:** Moisture content, water activity, TPC and radical scavenging capacities of red osier dogwood aerial parts extracted at different temperatures.

Sample	% Moisture content	Water activity	TPC	DPPH	TEAC	ORAC
Extraction at 75^o^C						
Raw material, 1 h	8.2 ± 0.4^gh^	0.43 ± 0.01^o^	8.53 ± 0.33^ef^	118.78 ± 9.79^e^	138.74 ± 36.41^bc^	129.99 ± 1.85^cd^
Spray dried extract, 1 h	6.6 ± 0.3^e^	0.25 ± 0.01^h^	16.81 ± 1.12^i^	266.06 ± 24.78^h^	184.99 ± 56.00^cd^	436.09 ± 12.25^f^
Spray dried extract, 2 h	5.2 ± 0.3^d^	0.19 ± 0.01^e^	14.72 ± 0.48^h^	254.22 ± 15.22^gh^	185.60 ± 41.61^cd^	426.27 ± 15.50^f^
Spent material, 1 h	1.6 ± 0.4^a^	0.11 ± 0.00^c^	5.48 ± 0.10^ab^	60.97 ± 5.53^a^	68.90 ± 2.66^a^	54.95 ± 6.01^a^
Spent material, 2 h	2.7 ± 0.6^b^	0.11 ± 0.01^c^	5.60 ± 0.13^ab^	57.03 ± 9.10^a^	52.85 ± 8.42^a^	65.07 ± 4.68^a^
Extraction at 85^o^C						
Raw material, 1 h	7.6 ± 1.2^fgh^	0.40 ± 0.00^n^	8.34 ± 0.52^def^	111.68 ± 1.00^de^	159.08 ± 3.37^c^	105.72 ± 2.86^b^
Spray dried extract, 1 h	7.5 ± 0.1^fg^	0.23 ± 0.00^g^	13.28 ± 0.15^g^	234.12 ± 5.29^g^	162.62 ± 23.97^c^	363.14 ± 15.23^e^
Spent material, 1 h	1.1 ± 0.5^a^	0.17 ± 0.01^d^	5.17 ± 0.04^a^	64.74 ± 3.27^ab^	57.25 ± 4.64^a^	61.22 ± 3.41^a^
Extraction at 95^o^C						
Raw material, 1 h	7.8 ± 0.5^fgh^	0.46 ± 0.00^p^	7.62 ± 0.11^cd^	115.39 ± 0.50^de^	157.47 ± 58.20^c^	125.76 ± 1.29^c^
Spray dried extract, 1 h	6.6 ± 0.5^e^	0.30 ± 0.00^j^	16.24 ± 1.05^i^	337.03 ± 18.88^j^	323.04 ± 68.13^e^	452.56 ± 12.43^g^
Spent material, 1 h	3.7 ± 0.6^c^	0.25 ± 0.00^h^	6.09 ± 0.08^b^	62.61 ± 1.27^a^	49.03 ± 3.81^a^	67.43 ± 2.04^a^
Extraction at 98^o^C						
Raw material, 1 h	9.3 ± 0.4^i^	0.60 ± 0.00^s^	8.97 ± 0.26^f^	142.88 ± 3.98^f^	185.43 ± 44.55^cd^	144.31 ± 2.16^d^
Spray dried extract, 1 h	6.7 ± 0.1^e^	0.29 ± 0.01^i^	21.80 ± 0.75^k^	452.94 ± 17.83^k^	551.59 ± 28.23^f^	528.95 ± 12.39^i^
Spent material, 1 h	1.7 ± 0.3^a^	0.10 ± 0.01^ab^	6.01 ± 0.08^b^	84.66 ± 3.88^bc^	100.69 ± 2.91^ab^	69.24 ± 4.74^a^

Results are presented as means ± standard deviation of triplicates. TPC – total phenolic content, expressed as g GAE/100 g sample, dry weight basis; DPPH – 2,2-diphenyl-picrylhydrazyl, expressed as mmol TE/100 g sample, dry weight basis; TEAC – Trolox equivalent antioxidant capacity, expressed as mmol TE/100 g sample, dry weight basis; ORAC – oxygen radical absorbance capacity, expressed as mmol TE/100 g sample, dry weight basis. GAE – gallic acid equivalent; TE – Trolox equivalent. Values in a column with same superscript letters are not significantly (p < 0.0001) different from each other.

Water activity of the raw materials, spray-dried extracts and freeze-dried spent materials ranged between 0.37-0.60, 0.19–0.32 and 0.10–0.25 respectively (Table 3). Generally, water activity values below 0.6 inhibit bacterial, yeast and mould growth as well as chemical and enzymatic changes in food (Clemenson et al., 2012; Fontana, 2007). With the exception of the raw material that was subjected to extraction at 98 °C which had water activity of 0.6, all the other raw materials had values below 0.5.

Generally, the spray-dried extracts had the highest TPC and all radical scavenging capacities (DPPH, TEAC and ORAC) followed by the raw materials, while the spent materials had the lowest values. This trend is in agreement with the phenolic composition as described above. There was an exception to this trend, in that, the TEAC values for spray-dried extracts prepared at 75 °C and 85 °C were not significantly (p > 0.0001) different from that of the raw materials. This indicates that hydrothermal extraction was more effective at 95 °C and 98 °C than at 75 °C and 85 °C. The agreement in trends between the phenolic composition and the TPC and radical scavenging capacities indicate that the phenolic acids and flavonoids identified in the ROD samples were the predominant compounds responsible for the radical scavenging capacities measured.

In comparing the extraction temperatures, it was observed that the TPC and ORAC values were highest for spray-dried samples extracted at 98 °C, followed by extraction at 95 °C and then 75 °C, while samples extracted at 85 °C had the lowest TPC and ORAC values. Similarly, DPPH and TEAC values were highest for sprayed-dried samples extracted at 98 °C, followed by those extracted at 95 °C. Samples extracted at 85 °C and 75 °C had comparable DPPH and TEAC values. This confirms that hydrothermal phenolic extraction of ROD was most effective at the highest temperature of 98 °C compared to all the other three (75 °C, 85 °C and 95 °C).

To put in perspective, TPC, DPPH, TEAC and ORAC values of spray-dried samples extracted at 75 °C, were 88%, 131%, 21% and 237% higher than that of their corresponding raw materials. On the other hand, TPC, DPPH, TEAC and ORAC values of spray-dried samples extracted at 98 °C, were 169%, 212%, 197% and 256% higher than that of their corresponding raw materials. It is worth noting that, although water is not the most ideal solvent for phenolic extraction (Al-Farsi and Lee, 2008), it is safer for use than organic solvents, especially when the target is for human or animal consumption, since organic solvents such as methanol and acetone are toxic. Heat applied during hydrothermal extraction therefore effectively disrupts the plant tissue matrix and facilitates release of the phenolic compounds (Sharma et al., 2015; Skotti et al., 2014).

As explained earlier, after the hydrothermal extraction, the spent materials retained some phenolic compounds (Table 2) that exhibited radical scavenging capacities (Table 3). This demonstrates that the spent materials can be used in developing high-value products such as antioxidant-rich animal feed (Wei et al., 2019).

### 3.3 Principal components analysis

**Figure 3:**
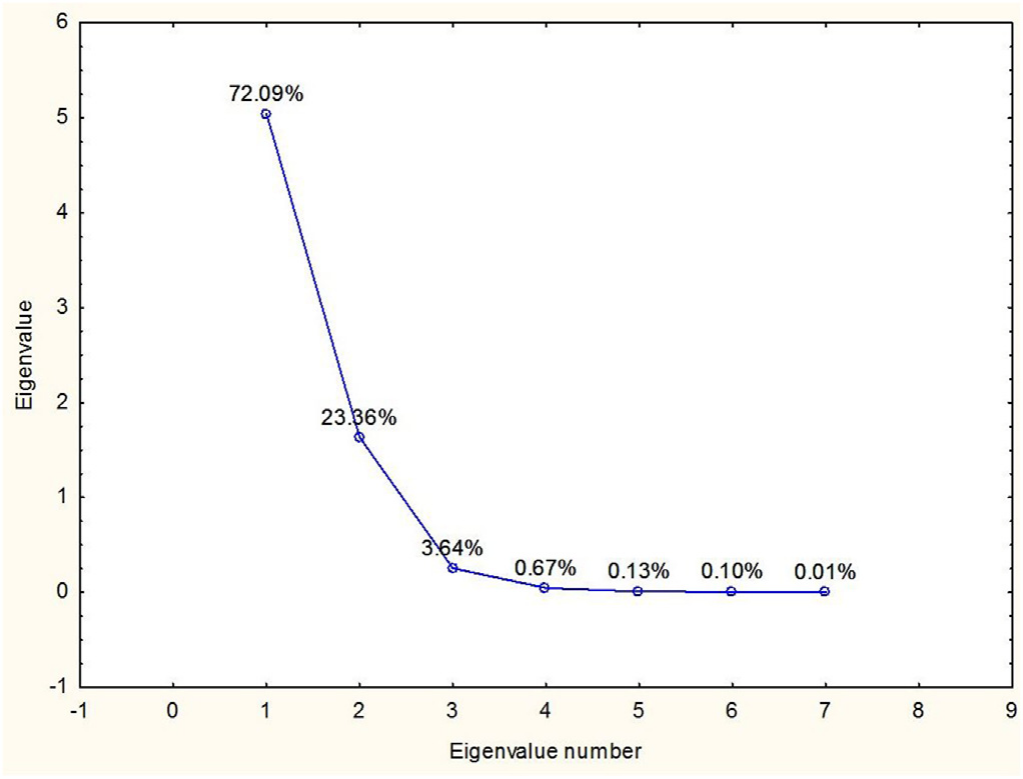
Scree plot showing Eigen values, principal components (Eigen numbers) and percentage of the total variance in the dependent variables for red osier dogwood.

Principal components analysis of the dependent variables, based on correlation matrix (Table 4), led to extraction of seven principal components, two of which had Eigen values higher than 1 (Figure 3). The first principal component accounted for 72% of the total variance in the dependent variables while the second principal component accounted for 23% of the total variance (Figure 3).

**Table 4:** Pearson correlation coefficient of moisture content, water activity, phenolics content and antioxidant activity of red osier dogwood.

	Moisture	Water activity	Total quantified phenolics	TPC	DPPH	TEAC	ORAC
Total quantified phenolics	0.434474	0.072096	1.000000	0.987851	0.978666	0.846316	0.989885
Moisture	1.000000	0.856559	0.434474	0.476732	0.448707	0.472426	0.422622
Water activity	0.856559	1.000000	0.072096	0.114345	0.115615	0.265148	0.030661
TPC	0.476732	0.114345	0.987851	1.000000	0.986363	0.877237	0.979950
DPPH	0.448707	0.115615	0.978666	0.986363	1.000000	0.924251	0.964364
TEAC	0.472426	0.265148	0.846316	0.877237	0.924251	1.000000	0.794437
ORAC	0.422622	0.030661	0.989885	0.979950	0.964364	0.794437	1.000000

**Figure 4:**
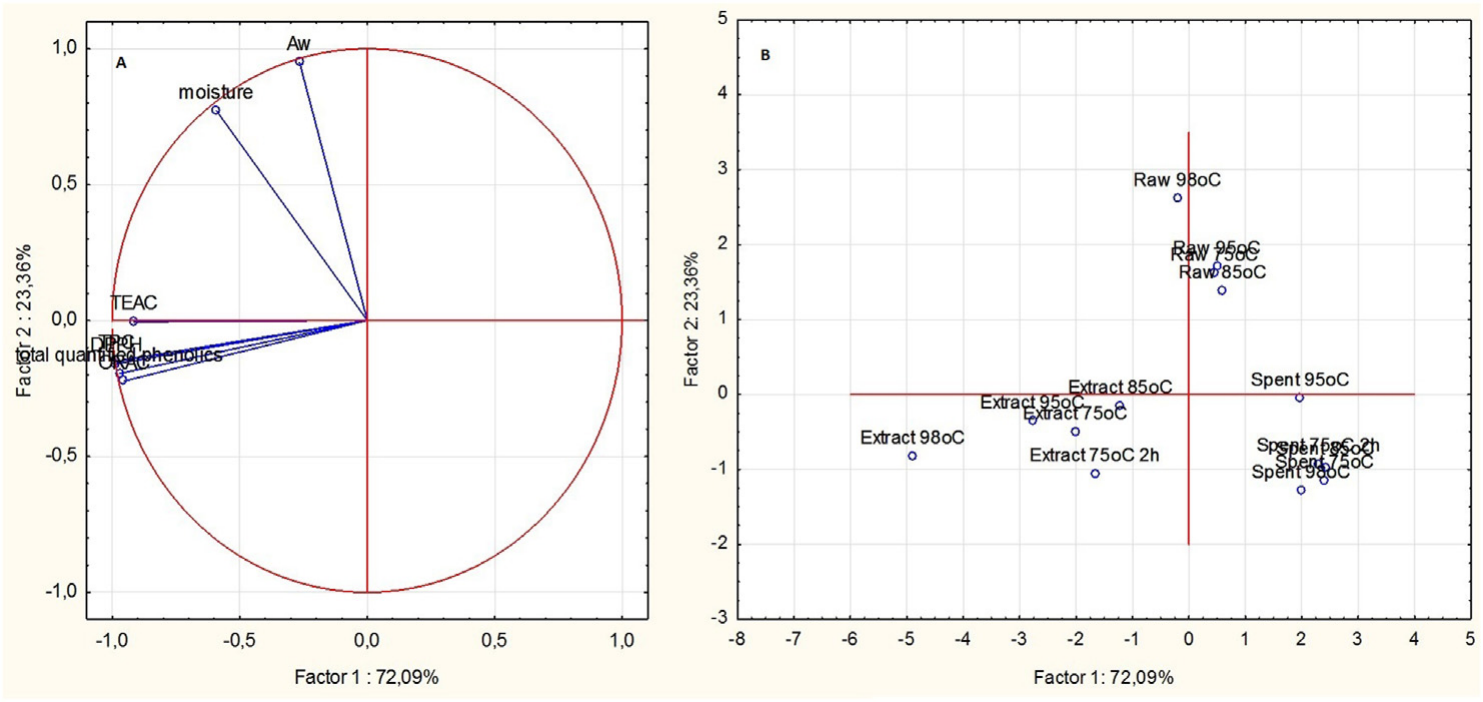
Principal components bi-plot of A: the dependent variables (moisture content, water activity, total quantified phenolic compounds, total phenolic content and antioxidant capacity) and B: the sample types of red osier dogwood leaves and stems.

The first principal component grouped the dependent variables together on the left axis of a bi-plot, while the second principal component separated the dependent variables into two groups, with moisture content and water activity grouped together, while total quantified phenolic compounds, TPC, and the radical scavenging capacities were also grouped together (Figure 4a).

The samples were also grouped into three clusters comprising raw materials, spray-dried extracts and spent materials (Figure 4b). The spray-dried extracts were clustered on the side of the horizontal axis similar to that of the total quantified phenolics, TPC and radical scavenging capacities, and this may be due to their relatively high levels compared to the raw materials and spent materials.

## 4 Conclusion

This research has demonstrated that constituent phenolic compounds of red osier dogwood dried leaves and stems, that exhibit antioxidant properties, can be extracted by hydrothermal treatment. The highest extraction temperature of 98 °C is the most effective in extracting phenolic compounds from the dried leaves and stems. All the raw materials, spray-dried extracts and spent materials have moisture content below 10% and water activity of 0.6 or lower, which are recommended for storing dried plant products. The spray-dried extracts therefore show promise for applications such as functional food ingredients and nutraceuticals. However, toxicological studies need to be done in order to confirm them as safe for human consumption, as well as defining dosage for use. The spent materials obtained after the extraction, retain some phenolic compounds that exhibit antioxidant properties. They can therefore find applications in high-value products such as animal feed.

## Declarations

### Author contribution statement

Franklin B. Apea-Bah: Performed the experiments; Analyzed and interpreted the data; Wrote the paper.

Dagmara Head: Conceived and designed the experiments; Performed the experiments; Wrote the paper.

Robert Scales, Ron Bazylo: Conceived and designed the experiments; Contributed reagents, materials, analysis tools or data.

Trust Beta: Conceived and designed the experiments; Contributed reagents, materials, analysis tools or data; Wrote the paper.

### Funding statement

This work was supported by the 10.13039/501100000038Natural Sciences and Engineering Research Council of Canada (Engage Grant) and the 10.13039/501100000196Canada Foundation for Innovation (New Opportunities Fund and Leaders Opportunities Fund).

### Competing interest statement

The authors declare no conflict of interest.

### Additional information

No additional information is available for this paper.
